# Case Report: Recessive Dystrophic Epidermolysis Bullosa With Severe Esophageal Stenosis: A Case Report and Literature Review

**DOI:** 10.3389/bjbs.2022.10200

**Published:** 2022-03-23

**Authors:** Zhen Xu, Tianqiao Huang, Min Pan, Yichuan Huang, Yan Jiang

**Affiliations:** ^1^ Deparment of Otolaryngology Head and Neck Surgery, The Affiliated Hospital of Qingdao University, Qingdao, China; ^2^ Deparment of Dermatology, The Affiliated Hospital of Qingdao University, Qingdao, China

**Keywords:** therapy, epidermolysis bullosa, recessive dystrophic epidermolysis bullosa, esophageal stenosis, dysphagia

## Introduction

Epidermolysis bullosa (EB) is a rare genetic disease that has no effective management or cure. Patients with EB may manifest with skin and mucous membrane fragility, blisters, erosions and scars. Based on the 2014 diagnosis and treatment guidelines, EB can be divided into four types: EB simplex (EBS), junctional EB (JEB), dystrophic EB (DEB), and Kindler syndrome (1). DEB usually affects the skin and nails at birth, which can be divided into two subtypes, namely the dominant dystrophic epidermolysis bullosa (DDEB) and recessive dystrophic epidermolysis bullosa (RDEB) (2). Furthermore, based on different clinical features, RDEB has been classified into severe Hallopeau-Siemens type (RDEB-HS) or the milder form called RDEB-nHS. Patients with RDEB-HS have systemic lesions and scars on hands and feet, leading to finger fusion and severe mucosal involvement, while those with RDEB-nHS may have local or systemic mild dermatological manifestations, mostly without finger fusion and without the involvement of the extradermal organs (3). Here, we report the case of a male patient with severe esophageal stricture due to recessive dystrophic EB. Currently, there is no effective treatment for EB complicated with severe esophageal stricture, although esophageal dilation and gastrostomy may be attempted.

## Clinical Case Presentation

A 28-year-old male was admitted to the Department of Otorhinolaryngology, Head and Neck Surgery of the Affiliated Hospital of Qingdao University, China, with a week history of progressively severe dysphagia to a liquid diet, hemoptysis, and sore throat for 1 week. Previously, he was diagnosed with COL7A1:DEB, Bart-type non-specific epidermolysis bullosa through genetic testing. He started experiencing irregular and intermittent hemoptysis with no hematemesis after swallowing pieces of shrimp skin 2 years ago, followed by a gradual onset of dysphagia. Since then, he had attended several hospitals for esophageal dilation or esophageal stent insertion, which had not been clinically successful due to the uncertainty of the therapeutic effect caused by congenital disease.

**FIGURE 1:**
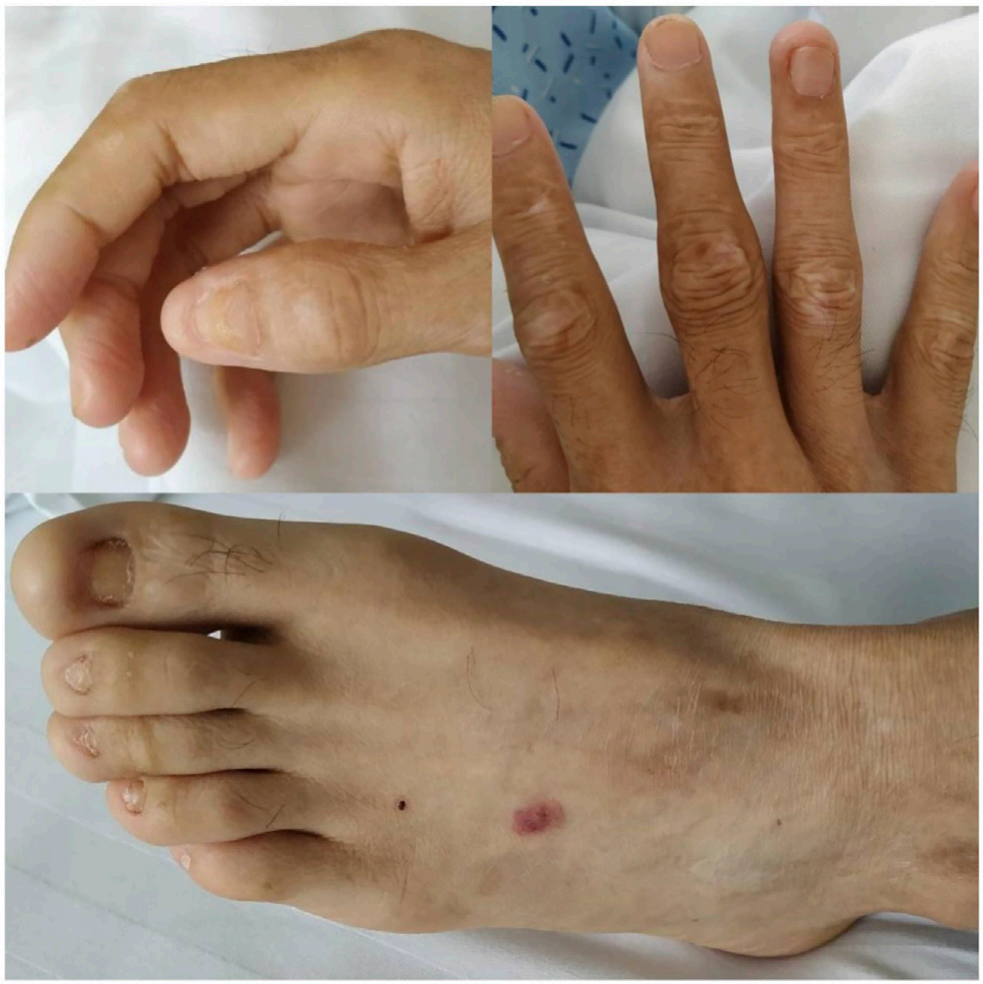
Clinical manifestations of the patient’s hands, feet, nails and skin: incomplete and deformed nails, skin scar.

**FIGURE 2:**
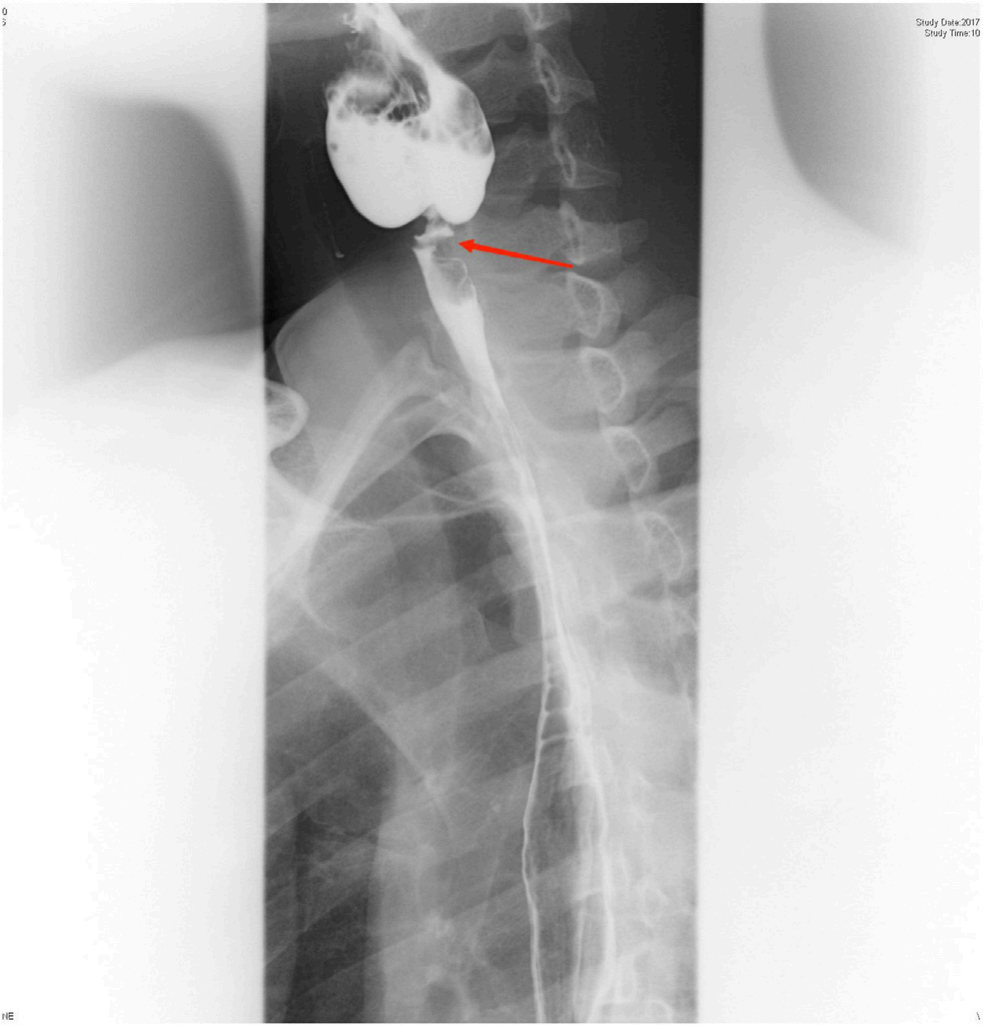
Esophagus barium swallow test: the proximal part of the esophagus appeared severely narrowed at the level of the C6 vertebral body, and the esophageal lumen proximal to the stenosis was significantly dilated.

**FIGURE 3:**
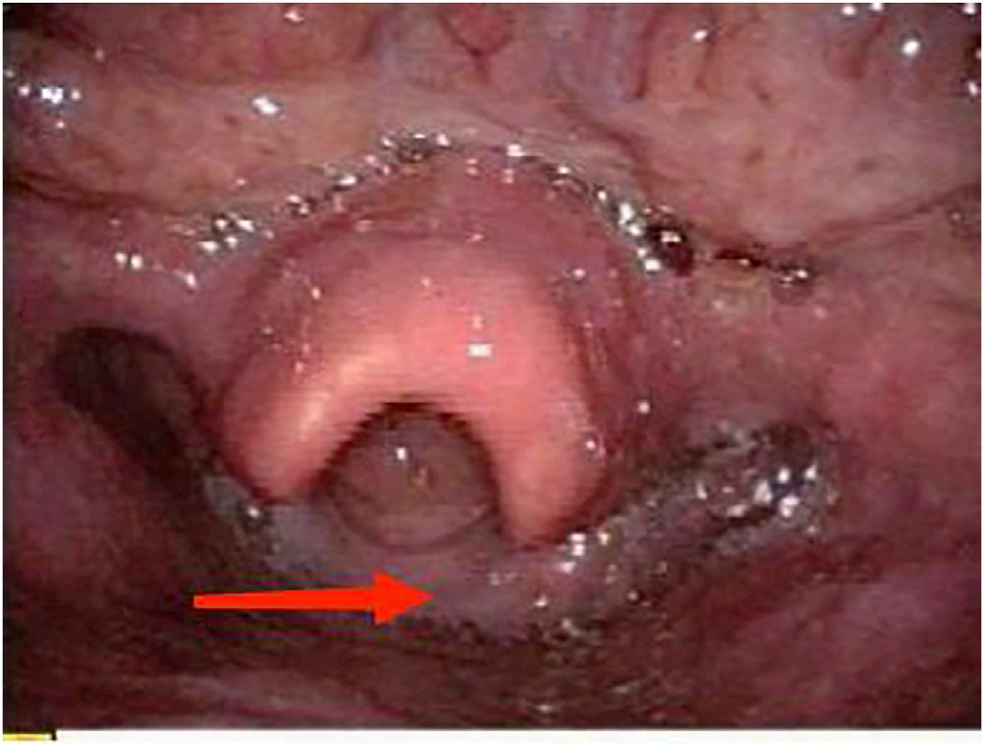
Electronic fiberoptic laryngoscopy: saliva accumulated in the hypopharynx, with thickened mucosa behind the scoop.

**FIGURE 4:**
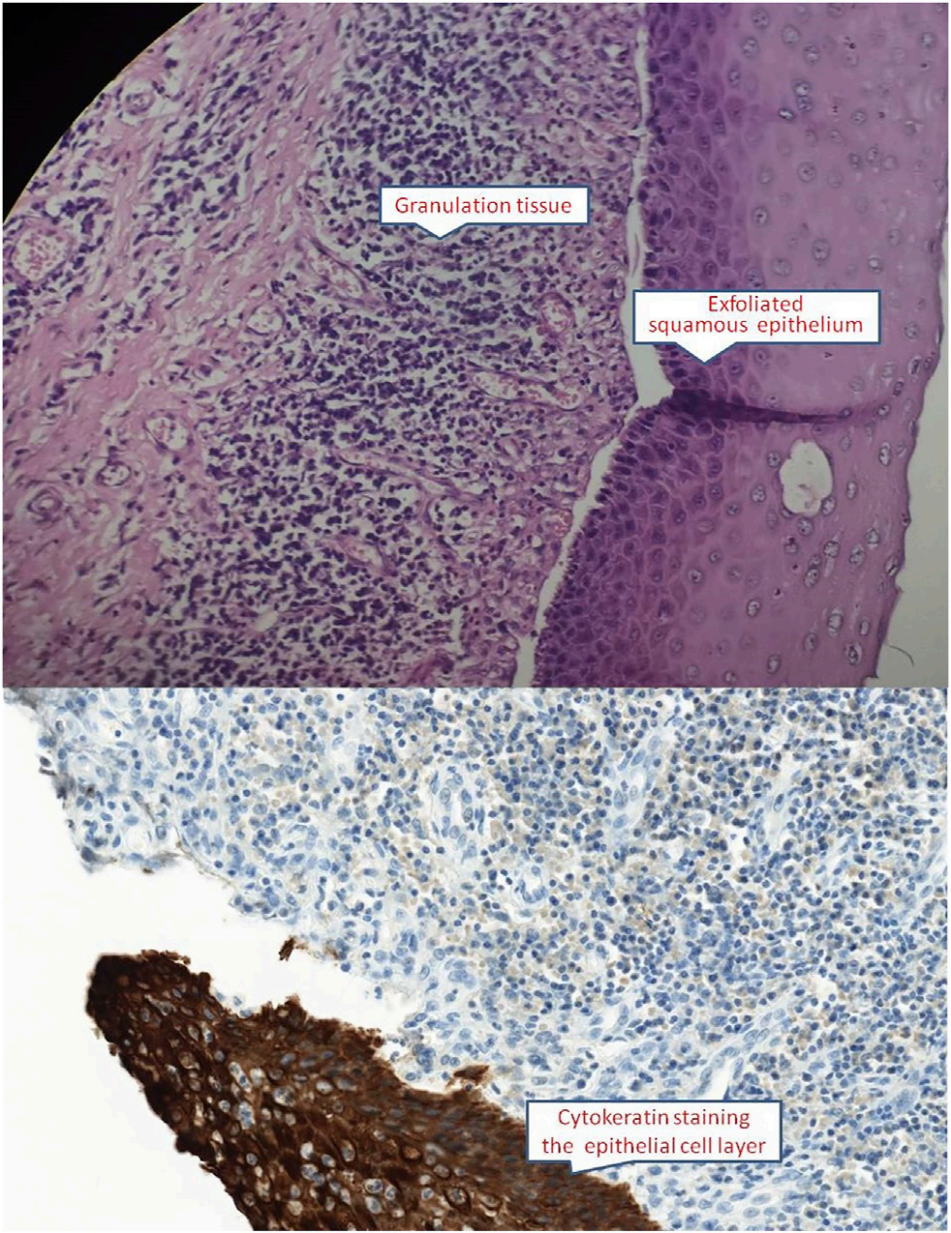
Esophageal mucosa showed squamous epithelial shedding and proliferation of submucosal granulation tissue (HE ×400 and CK immunocytochemistry ×400).

On physical examination, the patient weighed 53 kg, 170 cm in height, with a BMI of 18.34. Incomplete and deformed nails on hands and feet were observed (Figure 1). Based on the Atkinson score (4), the patient’s dysphagia was grade IV (Grade 0: No dysphagia and able to eat normally; Grade I: Occasional dysphagia but able to eat some solid food; Grade II: able to consume a semi-fluid diet; Grade III: able to consume liquid diet only; grade IV: complete dysphagia. The nutritional assessment revealed that the patient had a low body mass index with moderate malnutrition. He was investigated with a barium swallow test (Figure 2) and electronic laryngoscopy (Figure 3), which demonstrated a severely narrowed hypopharynx-esophageal entrance. The strictured esophagus could not be traversed even with an ultra-fine gastroscopy. Biopsies were taken from the strictured esophagus, which showed squamous epithelial shedding and proliferation of submucosal granulation tissue (Figure 4).

According to the patient’s previous genetic sequencing (supplied by the Beijing Kangso Medical Inspection), he had COL7A1:DEB, Bart-type non-specific epidermolysis bullosa (detection gene: COL7A1 [type VII collagen gene], position: chr3:48606879, variation: c.7012C>T and c.7615G>T, variation type: heterozygous), which belongs to an extremely rare autosomal recessive genetic dystrophy type of epidermolysis bullosa (RDEB). Further genetic testing on the patient’s immediate family members (father, mother, and elder brother) revealed that the patient carried two heterozygous mutation vectors, with c.7012C>T derived from his father’s heterozygous gene. c.7012C>T (nucleotide 7012 in the coding region is changed from C to T) represents the heterozygous nucleotide mutation, which causes the codon containing nucleotide 2338, coding for arginine, to be read as a stop codon (p.Arg2338Ter), leading to premature termination of the peptide chain synthesis, which is thus a nonsense mutation. c.7615G>T was derived from his mother’s heterozygous gene, while the gene type of the patient’s brother at this locus was similar to that of the mother. c.7615G>T (nucleotide 7615 in the coding region is changed from G to T) represents the heterozygous nucleotide mutation, which causes the amino acid 2539 to change from glycine to cysteine (p.Gly2539Cys), and is recognized as a missense mutation. Mutations in the COL7A1 gene can lead to the abnormal synthesis of type VII collagen. We postulated that in our patient, the c.7012C>T and c.7615G>T gene mutations might have affected the protein function.

## Discussion

Dystrophic epidermolysis bullosa can be inherited in an autosomal dominant or recessive pattern. Patients with DEB may present with recurrent skin blisters, ulcers, atrophic scars, miliary rash, mucosal lesions, or white papules. Micro-dense horizontal tissue separation under the basement membrane, skin blisters that develop at birth or in infancy decrease in frequency with age, and family variation (5). Following a mild mechanical injury, blisters, erosions and scars of the skin and mucous membranes are formed. In the pathogenic gene COL7A1, c.7012C>T is derived from a heterozygous mutation of the father, while c.7615G>T is derived from a heterozygous mutation of the mother. After searching online databases (http://www.col7a1-database.info), It was found that the pathogenicity of the variant c.7012C>T associated with epidermolysis bullosa had been previously reported. However, the variant c.7615G>T, which is extremely rare clinically, has not been linked to the disease. In our patient, a detailed inquiry in the medical history revealed that the patient had gingival mucosal blisters since birth, which was apparent with ruptures during feeding. Repeated episodes of these had continued during infancy and toddler age, which eventually resolved at adulthood. However, nail dystrophy such as abnormal nail shape and slow growth had persisted. Furthermore, recurring blistering and ulceration of the esophageal mucosa due to frictions with swallowed objects had eventually led to the occurrence of esophageal stenosis. Although the incidence of esophageal stenosis in patients with DEB is more than 50%, reports of patients with severe stenosis are rather limited in the literature (6). To date, there is no effective treatment for epidermolysis bullosa. In general, skin care affects the prognosis and quality of life of patients (6). Nevertheless, several studies have suggested the use of drugs to improve skin conditions in patients with EB. For instance, losartan (7) may relieve skin blisters, raloxifene reduces fibrosis of the skin and mucous membrane, while N-acetylcysteine reduces skin inflammation (8). Bornert et al. have shown that QR-313 (an antisense oligonucleotide) regulates the splicing of exon 73 of type VII collagen, thus restoring the abundance of collagen VII in the skin of RDEB patients and improving the quality of wound healing by influencing the fibrosis of the wound, thereby reducing the formation of wound scars and recurring skin damage (9). Treatment strategies to restore the functional expression of defective proteins including *in vivo* and *in vitro* gene therapy, protein replacement therapy, cell therapy, and pharmacological methods are currently in the clinical trial stage (10).

For mild to moderate esophageal stenosis as a result of common etiology, endoscopic esophageal dilatation is currently the preferred treatment (11). The mucosa of the lesion is removed by endoscopic esophageal stenosis and mucosal peeling, followed by steroid or mitomycin injection into the mucosa. Mycin C is used to reduce the risk of scar re-proliferation. while esophageal stent can also be considered. With time, the fragility of the esophageal mucosa increases, which can be further exacerbated with deteriorating stenosis as a result of esophageal dilation and esophageal stent implantation. At present, there is no effective treatment to halt the further development of esophageal inflammation (12). Our patient had not received any effective treatment for 2 years after the onset of the disease, leading to eventual severe esophageal stricture. Recently, Koklu et al. have described the use of choledochoscopy in anterograde-retrograde endoscopy to dilate completely the narrowed esophagus in patients with RDEB-related esophageal stenosis, though the long-term outcome of this procedure remains unclear (13). For severe esophageal stenosis, a gastrostomy may be the ultimate treatment for patients with RDEB (6), given the lack of effective treatment options currently for this genetic disease with progressive severity.

DEB is a rarely seen in the clinic. Through relevant tests and investigations, we have identified ways in which to ameliorate the clinical symptoms, nevertheless there is no effective cure for this type of disease. The means of effecting a complete cure remains a challenge for future investigations.

## Data Availability

The original contributions presented in the study are included in the article/Supplementary Material, further inquiries can be directed to the corresponding author.
